# The forgotten foe returns: cholera’s warning to a complacent world demanding urgent action

**DOI:** 10.1097/MS9.0000000000005361

**Published:** 2026-07-08

**Authors:** Shamikha Cheema, Haleema Sadia, Muhammad Talha, Muhammad Rafay-Ur-Rehman, Waseem Sajjad, Ihsan Qamar

**Affiliations:** a Department of Medicine, King Edward Medical University, Lahore, Pakistan; b Department of Medicine, Fazaia Ruth Pfau Medical College, Air University, Karachi, Pakistan; c Department of Medicine, Nishtar Medical University, Multan, Pakistan; d Department of Medicine, Spinghar University, Kabul, Afghanistan

**Keywords:** cholera, cholera outbreak, outbreak response, public health infrastructure, WHO

## Abstract

Cholera, a deadly bacterial infection, affects 1.3–4 million people annually. Reports in early 2025 show a sharp rise in cases, especially in the Eastern Mediterranean and African regions, posing a serious global threat. This perspective argues that this reappearance is not just a periodic outbreak, but rather the result of interconnected global crises: climate change, armed conflicts, and a fragile healthcare system. This article analyzes these factors driving the spread of cholera and critically evaluates the roles of the World Health Organization (WHO) and other international bodies. While WHO measures have proven valuable, this article underscores the urgent need for stronger public health systems, early detection, and improved testing capacity to withstand future outbreaks. Enhancing surveillance, supporting vaccine and treatment development, and fostering cross-border cooperation are key to preventing regional spread. A coordinated global response involving governments, international organizations, and Non-Governmental Organizations is essential to curbing the crisis. Priority actions include investing in WASH (water, sanitation, and hygiene) infrastructure, expanding oral cholera vaccine production, and developing climate-resilient health systems. Cholera remains a preventable disease, but only through collective and adaptive action can its resurgence be halted and global health security safeguarded.

## Introduction

Cholera, an acute diarrheal infection caused by consuming food or water contaminated with the bacterium vibrio cholerae, annually affects approximately between 1.3 and 4 million people, causing 143 000 deaths^[^1,2^]^. It is characterized by fatal, severe acute watery diarrhea, vomiting, and rapid dehydration^[^1^]^. Cholera has been persistent in countries with less access to safe water, sanitation, and facilities for hygiene. Moreover, the rapid transmission and tendency to cause hypovolemic shock highlight the urgency for surveillance and proper measures^[^2^]^. These outbreaks continuously pose a public health threat. This short communication analyzes the recent expanding burden of the cholera outbreak, asserting that its reappearance is a direct result of the co-occurrence of climate change, armed conflict, and a systemic weakness in the global health infrastructure. We critically analyze the current outbreak hotspots and factors driving the outbreak, along with the global context. This work has been reported in accordance with TITAN guidelines^[^3^]^.

HIGHLIGHTS

- Cholera cases surged in early 2025, especially in the Eastern Mediterranean and African regions.
- Stronger public health systems and early detection are crucial for outbreak control.
- Surveillance, vaccine development, and cross-border cooperation can curb regional spread.
- Addressing poor sanitation, unsafe water, and weak health systems is essential.
- A coordinated global response from governments, the World Health Organization, and Non-Governmental Organizations is urgently needed.

### Current outbreak hotspots

From January 1 to 29 December 2024, a total of 804 721 cholera cases and 5805 deaths were reported globally across five WHO regions, with the Eastern Mediterranean Region being the region with the highest reported cases^[^4^]^. From 1 January to 23 February 2025, the African region has recorded the highest reported cases of cholera (70 488 cumulative total cases and 808 deaths). Currently, within the African region, South Sudan, the Democratic Republic of Congo, and Angola are facing particularly severe outbreaks. In the Eastern Mediterranean region, Afghanistan, Sudan, and Yemen are facing this issue. Particularly, while Myanmar has reported a high number of cases in the Southeast Asian region, the absence of reported deaths shows heterogeneity in outbreak severity and response capacity significantly^[^5^]^.

## Current global statistics

In the period between 1 January and 23 February 2025, a report of 70 488 cholera cases with 808 associated deaths spanning 23 countries across three WHO regions was made^[^5^]^. A critical analysis of these recent statistics reveals a concerning trend. While the 70 488 cases reported in early 2025 show a slight fall from the 79 510 cases in the same period of 2024^[^6^]^, this disguises a 32% increase in reported deaths in February 2025 compared to January 2025^[^4,7^]^. This increase in the case-fatality rate suggests that health systems are unable to cope, and the severity of the outbreaks is becoming worse. The persistence of such huge outbreaks, especially in the African and Eastern Mediterranean Regions, shows that the cholera outbreak globally remains a Grade 3 emergency. This continuous crisis, despite the monthly or yearly variations, is driven by long-term factors and demands urgent international action. We selected data from authoritative sources, including the WHO Multi-country Cholera Upsurge External Situation Report #24, as summarized in Table 1^[^5^]^.

**Table 1:** Cholera and acute watery diarrhea cases and deaths from 1 January 2025 to 23 February 2025.

Region (WHO)	Country/area	Confirmed cases	Deaths	CFR^a^ (%)
Africa	South Sudan	18 031	243	1.3
	Democratic Republic of Congo	8401	171	2.0
	Angola	4914	180	3.7
	Ghana	1895	12	0.6
	United Republic of Tanzania	1762	13	0.7
	Nigeria	1113	28	2.5
	Mozambique	298	0	0.0
	Zambia	277	9	3.2
	Ethiopia	177	3	1.7
	Togo	159	4	2.5
	Zimbabwe	144	2	1.4
	Burundi	86	0	0.0
	Uganda	80	1	1.2
	Malawi	69	1	1.4
Eastern Mediterranean	Afghanistan	12 661	5	0.0
	Yemen	9784	9	0.1
	Sudan	5479	126	2.3
	Pakistan	2816	0	0.0
	Somalia	1409	1	0.1
Southeast Asia	Myanmar	771	0	0.0
	Bangladesh	79	0	0.0
	Nepal	77	0	0.0
	Thailand	5	0	0.0


^a^ CFR, case fatality rate. It is the proportion of the number of deaths divided by the number of confirmed cases of a disease.

## Key factors driving the outbreak

One of the main factors driving the cholera epidemic is poverty and the higher density of populations among these countries, especially in the African region, which limits access to clean water, proper sanitation, and required healthcare services^[^8,9^]^.

A primary catalyst is human conflict, which dismantles the foundational systems necessary for public health. A central part of this crisis is the direct and indirect effects of armed conflicts, especially in the African and Middle Eastern regions. Conflicts of this nature cause deliberate or collateral destruction of WASH (water, sanitation, and hygiene) and healthcare infrastructure, causing massive populations to be displaced into overcrowded areas like refugee camps, which become high-risk grounds for cholera^[^9^]^. Case studies from Sudan and Yemen offer distinct but interpretive examples, both of which were prominent in the recent surge (Table 1). In Sudan, conflict-driven displacement has forced the population into camps with barely existing sanitation and clean water. In Yemen, the conflict has directly caused the destruction of established water infrastructure. Moreover, the collapse of the healthcare system and malnutrition have increased the chances of fatal outcomes^[^9^]^. Similarly, Yemen’s conflict has also destroyed the infrastructure for clean water and sanitation, leaving a large part of the population dependent on unsafe water sources. This collapse of infrastructure, combined with a disrupted healthcare system and increasing rates of acute malnutrition, has caused one of the largest cholera epidemics in modern history^[^8^]^.

Climate change poses another threat: the rising temperatures, changing rainfall trends, and extreme weather changes contribute to conditions such as droughts, floods, and contamination of the sources of water^[^8,9^]^. Bekele *et al* highlight the significant role of climate change in exacerbating cholera outbreaks in Africa, affecting water sources through droughts and floods^[^10^]^. Adriano *et al* illustrate this with the devastating effect of Cyclone Freddy on the cholera outbreak in Malawi^[^11^]^.

As the COVID-19 pandemic surfaced, it shifted the focus away from the cholera cases, thereby

disrupting the public resources and impacting the cholera control measures, such as surveillance^[^9^]^. Uwishema *et al* underscore how the COVID-19 pandemic created a double burden on Africa’s health systems, hindering cholera response efforts^[^12^]^.

## The need for urgent action

Cholera spread remains a potentially serious global health crisis, with more than 70 488 cholera cases and 808 deaths reported from 23 countries alone between January and February 2025^[^4^]^.

This means there was a sudden jump in fatalities, as cholera-related deaths increased by 32% in February compared to January^[^4,7^]^. Vulnerable populations suffer disproportionately in conflict zones, floods, and poorly supplied regions. For instance, there were 7 119 reported cases of cholera, with 258 deaths in the first quarter of 2025 in Angola alone, compared to no reported cases in 2024 during the same period^[^13^]^.

### Access to clean water and sanitation

Although the cholera epidemic is directly promoted by a lack of clean water and sanitation, this deficiency is not an isolated issue; rather, it is a result of deeper, upstream drivers. This breakdown of WASH infrastructure is usually a consequence of poverty, armed conflicts, and political instability, which must be addressed for a long-term solution to be proposed^[^9^]^. After the year 2000, over 2 billion people worldwide have been provided with unsafe drinking water, and this circumstance leaves a good ground for cholera transmission^[^14,15^]^. Investments in WASH infrastructures have shown a significant reduction in cholera incidence. Chlorination of water supplies, along with hygiene promotion campaigns, has reduced transmission risks during previous cholera outbreaks. Long-lasting prevention of cholera requires community strengthening and maintenance of water systems, which must be preserved at all costs. Additionally, the integration of WASH strategies with vaccination campaigns will further enhance benefits in outbreak control and resilience^[^15^]^.

### Strengthening healthcare systems

Timely access to healthcare services is crucial in mitigating the effects of cholera mortality. Prompt administration of ORS and intravenous fluids can reduce case fatality rates below 1%, but many areas affected face serious obstacles to accessing healthcare. In Africa, for instance, high rates of “community deaths,” or deaths occurring outside health facilities, reinforce the pressing need for better healthcare delivery systems. There is a need to reinforce community-based care systems, coupled with mobile health units. Training local health professionals would ensure rapid detection and response to outbreaks in remote or underserved areas^[^4^]^.

## Global response and cooperation

### Role of international organizations

The role and limitations of international organizations like the World Health Organization (WHO) are crucial in coordinating responses to cholera outbreaks. A Health Emergency Appeal by WHO for 2025 requires funding of $45.6 million for cholera control activities all over the world^[^15^]^. Another WHO recommendation emphasizes multi-sectoral collaboration for improving preparedness and response. This encompasses bolstering surveillance systems, developing new WASH structures, and ensuring that oral cholera vaccines reach those in need in good time. WHO also supports countries in building rapid laboratory detection and confirmation of cholera outbreaks. Boosting international solidarity, technical support, and policy guidance to minimize the spread and impact of cholera are among WHO’s global leadership functions, highlighting current response efforts and recommendations^[^16^]^. However, the effectiveness of these organizations is often limited by funding gaps and the political will of the member states. Anis *et al* discuss the multi-country cholera outbreak alert in Kenya, highlighting current response efforts and the continuous challenges in their implementation on the ground^[^16^]^.

### Challenges in oral cholera vaccine supply

Oral cholera vaccines (OCVs), such as Shanchol, Dukoral, and Euvichol-Plus, have played an important role in managing the spread of cholera; however, the demand usually outruns the supply. February 2025 witnessed the stabilization of vaccine production, averaging stockpiles of 5.5 million doses per month, thus meeting the emergency threshold but not the ever-growing global need^[^4,14^]^. With only one global supplier, EuBiologics is expected to produce 70 million doses in 2025, whereas countries this year requested 74 million doses. The gap remains critical^[^15^]^. This shortage has caused the WHO to temporarily suspend the standard two-dose vaccination strategy and favor a single-dose approach, which is a practical step to maximize availability, but offers only a short-term solution. Since 1997, over 73 million doses have been distributed through international coordination (WHO, UNICEF, Médecins Sans Frontières) to 23 countries, but vaccine supply constraints persist due to complex production processes, low commercial incentives, and fluctuating demand. To address shortages, there is a pressing need to scale up production capacity by adding more large-scale manufacturers beyond EuBiologics, as the current single-supplier model limits supply flexibility and responsiveness^[^17^]^.

### Government and NGO contributions

Strengthened surveillance systems and investments in preparedness will motivate governments to drive national initiatives. An example is hygiene monitoring to collect real-time data on cholera cases, leading to more efficient resource allocation. Beyond these national initiatives, Non-Governmental Organizations (NGOs) need to educate communities in preventive practices – such as handwashing and safe practices related to food – and facilitate the distribution of clean water and sanitation kits in the most vulnerable areas. The engagement of local organizations is equally important, as it promotes deep community trust to navigate local contexts efficiently. Funding these local partners is essential to maintain the sustainability of interventions, as they ensure that health interventions are accepted and maintained long after the international teams leave. Collaborations between governments, NGOs, and international health agencies can ensure continued funding and technical support. Such partnerships are important for building the resilience of communities against outbreaks of cholera. Anis *et al* also provide recommendations for strengthening surveillance systems and community interventions in the context of the Kenyan outbreak^[^16^]^.

Furthermore, reinforcing surveillance systems in the 21st century requires adapting to new technologies. Newly surfacing tools like large language models (LLMs) offer the analysis of vast datasets from various sources, such as social media and news reports, in order to allow the early detection of outbreaks and enhance resource allocation. However, a global-scale analysis by Lyu *et al* shows a significant gap: the application of LLMs in public health is still underdeveloped compared to other medical fields. There is an urgent need to drive these tools toward addressing global health crises like cholera. Furthermore, the study also warns about an increased risk of global health inequalities in resource-poor regions, which are the epicenters of outbreaks like cholera, due to the least access to and benefit from these tools^[^18^]^.

## Conclusion

The current worldwide crisis of cholera, aggravated by a combination of poor infrastructure for WASH, climate change, and fragile health systems, calls for immediate, coordinated action by government agencies and international organizations, in conjunction with NGOs. The comprehensive approach must also tackle the underlying causes, such as conflict and poverty. It should also address the challenges faced in the supply chain by increasing OCV production and ensuring equitable access to preventive measures. Empowering local organizations must also be considered a fundamental part of this strategy to ensure sustainable solutions. Governments must give priority to cholera control; international organizations must provide the financing for the program; and NGOs must expand community interventions to combat this outbreak.

## Data Availability

The data supporting the findings of this study are available within the article’s reference section. No additional datasets were generated or analyzed during the current study.

## Peer and provenance statement

Not commissioned; externally peer-reviewed.
